# Observation on clinical effect of Huoxue-Jiangtang decoction formula granules in treating prediabetes: a randomized prospective placebo-controlled double-blind trial protocol

**DOI:** 10.1186/s12906-022-03755-2

**Published:** 2022-10-19

**Authors:** Peng-Xiang Zhang, Lin Zeng, Lu Meng, Hui-Lin Li, Heng-Xia Zhao, De-Liang Liu

**Affiliations:** 1 The Fourth Clinical Medical College of Guangzhou University of Chinese Medicine, Shenzhen, Guangdong People’s Republic of China; 2 Shenzhen Traditional Chinese Medicine Hospital, Shenzhen, Guangdong People’s Republic of China; 3 Endocrinology Department of Shenzhen Traditional Chinese Medicine Hospital, No. 1 Fuhua Road, Shenzhen, 518033 Guangdong Province People’s Republic of China

**Keywords:** Prediabetes, Huoxue Jiangtang formula, Randomized controlled trial, Traditional Chinese medicine

## Abstract

**Background:**

Prediabetes is a hypermetabolic syndrome with blood sugar levels falling between the normal and diabetes. People with prediabetes have a significantly increased chances of developing diabetes, cardiovascular and cerebrovascular diseases, tumors, dementia, and other diseases in the future when compared to the healthy population. However, prediabetes is mainly treated based on lifestyle intervention, currently without targeted drug treatment plan. Traditional Chinese medicine (TCM), which has a longstanding experience, has been shown in clinical studies to be effective for the treatment of diabetes and its related complications. Furthermore, different dosage forms such as decoction and granule have developed gradually in clinical application. Preliminary studies have found that Huoxue-Jangtang Decoction (HJD), with good hypoglycemic and lipid-regulating effects, is potentially one of the complementary and alternative treatments for prediabetes. Therefore, this project intends to perform a prospective clinical study to observe the clinical effectiveness of HJD on prediabetes and the consistency of the efficacy of formula granules and the elixation.

**Methods:**

This is a prospective, randomized, double-blind, and placebo-controlled clinical trial. A total of 183 participants are randomly assigned to HJD Formula Granules plus lifestyle intervention, HJD Elixation plus lifestyle intervention, and placebo plus lifestyle intervention. All subjects undergo 1 day of screening before participating in the study, followed by 84 days of drug intervention and observation. During and after treatment, the main outcome measures include fasting blood glucose and 2-hour postprandial blood glucose.

**Discussion:**

This research attempts to verify the clinical efficacy and possible mechanism of HJD in the treatment of prediabetes, and prove the consistency of HJD Formula Granules with HJD Elixation. This study also aims to provide a treatment that is both effective and safe for prediabetic patients.

**Trial registration:**

ClinicalTrials.gov Identifier: ChiCTR2200060813, Registered 12 June 2022.

## Background

Prediabetes, considered to be an important clinical stage of diabetes, is a hypermetabolic syndrome with blood sugar levels falling between the normal and diabetes. In the past 40 years, the lifestyle and diet structure of Chinese people have undergone great changes due to a rapidly expanding economy. A lack of exercise and high-calorie diet have significantly increased the proportion of prediabetic and diabetic patients in the Chinese population. The epidemiological statistics in China in 2017 show that 35.2% of the population have pre-diabetes, and 11.2% of the population have diabetes. However, only 4.0% of them have been clearly diagnosed just 49.2% of which have received adequate treatment [1, 2].

When the prediabetic patients do not receive timely diagnosis and treatment, about 5–10% of them will rapidly progress to the stage of diabetes, and the rest will gradually develop diabetes over the next 5 years. In the meantime, the incidence and severity of diseases such as coronary artery disease, heart failure, hypertension and retinopathy in patients with prediabetes are aggravated with more obvious blood glucose fluctuation, thus seriously affecting their quality of life [3–6]. As a result, timely diagnosis and intervention for diabetics can postpone the progression of prediabetes to diabetes and lower the risk of related complications, which is critical for both society and patients.

Fat, a strong temptation for human beings, provides necessary calories for normal physiological activities as one of the important food types. In a meta-analysis of dietary behavior, it is found that Chinese patients with type 2 diabetes generally preferred fat, oil, sweets and meat [7]. Long-term high-fat food intake can reduce an individual’s sensitivity to fat, leading to excessive fat intake, weight gain and insulin resistance [8]. Several studies have also discovered that people who consume a high-fat diet are at a markedly increased risk of experiencing prediabetes [9–12]. For this reason, the American Diabetes Association (ADA) recommends that diabetes and prediabetes patients should adjust their lifestyle and choose whole grains and fiber over high-fat, high-calorie and high-sweetness food to reduce the risks associated with diet [13].

The Diabetes Prevention Program has shown that lifestyle intervention can decrease the occurrence of prediabetes conversion by 58% [14]. In addition, lifestyle interventions require no additional financial costs or time requirements, so patients can do corresponding exercise in their free time, which remains the main measure and first choice for the prevention and treatment of prediabetes [15–17]. However, it’s not easy for some people to change a long-standing diet or lifestyle, and the lack of a consistently effective lifestyle intervention is also less effective in prediabetes. Although clinical researches have found that metformin, acarbose, orlistat and other drugs can prevent prediabetes from developing into diabetes by reducing body weight, improving glucose metabolism and other mechanisms, etc., there is still no consensus [18–21].

Traditional Chinese medicine (TCM) has been used to treat a wide range of diseases in China for thousands of years. In the process of application, granules, powder and other new formulations have been developed, however, formula granules and elixation are the two most widely used dosage forms. Previous research has found that TCM therapies can noticeably and securely improve the symptoms and clinical indicators of diabetes and prediabetes patients, so it appears to be a potential complementary therapy for prediabetes [22, 23].

In a meta-analysis of 26 randomized controlled trials, TCM treatment delayed the development of impaired glucose tolerance to diabetes and increased the likelihood of normalizing blood glucose in diabetics compared to the control groups. Compared with lifestyle intervention alone or drug therapy alone, TCM therapy had a more significant effect on improving the symptoms and indicators of prediabetes, diabetes and related complications [24].

Huoxue-Jangtang Decoction (HJD) is the precompounded prescription order of the Endocrinology Department of Shenzhen Traditional Chinese Medicine Hospital (Shenzhen TCM Hospital). Our earlier studies have shown that HJD has the effects of lowering glucose and controlling lipid, improving insulin resistance and reducing oxidative stress reaction, etc. It has been clinically applied to the treatment of T2DM, diabetic nephropathy, diabetic peripheral vascular disease and other diseases, with good therapeutic effect and safety [25–27].

However, research on the efficacy and clinical adverse reactions of HJD in prediabetic patients is still lacking. In addition, while the decoction is effective, the preparation process is tedious and time-consuming. Granules can be taken after mixed with warm boiled water, which can bring great convenience to patients, but there still lack persuasive evidence of efficacy consistency between the two dosage forms. For this reason, we designed this study protocol to evaluate the effectiveness and tolerability of HJD in the prevention and treatment of prediabetes. In addition, it is also aimed to verify the consistency between the Elixation of Huoxue-Jiangtang Decoction (HJD Elixation) and the Formula Granules of Huoxue-Jiangtang Decoction (HJD Formula Granules).

## Methods/design

### Design and setting

This study is a prospective, randomized, double-blind, placebo-controlled trial that has been registered with the China clinical trials registry (registration number: ChiCTR2200060813). All the documents including study protocol, informed consent, and Case Report Forms (CRF) meet the requirement of the Helsinki declaration, and have been reviewed by academic committee and ethics committee of Shenzhen TCM Hospital. This protocol was compiled in line with the SPIRIT 2013 (Table 1).

**Table 1:**
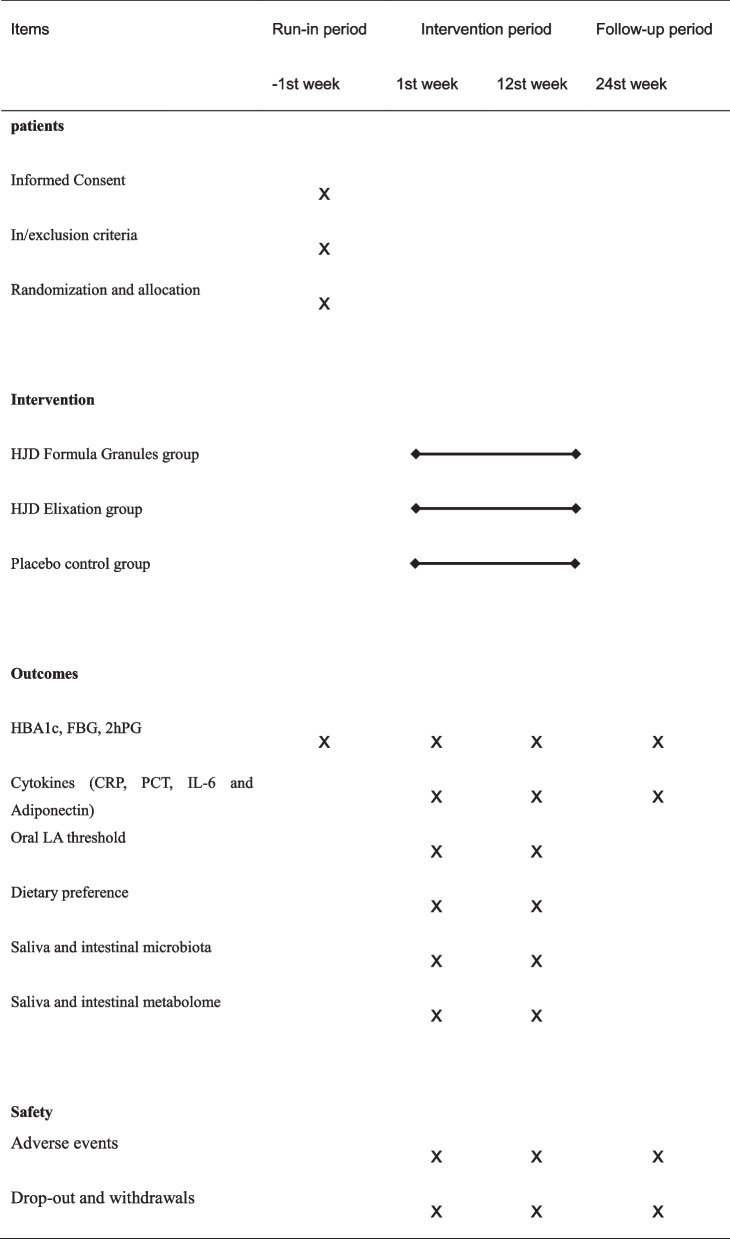
A standard protocol items: recommendation for interventions for trials (SPIRIT)

### Participants

#### Sample size

Sample size is calculated by PASS 15.0 software, with the significance level of 0.05. A total of 189 cases (63 cases in each group) are required to detect inter-group differences in 2.6 mmol/L postprandial 2 h glucose, with a statistical power of 90%. The sample size calculation assumes a standard deviation of 2.0 mmol/L and a shedding rate of 20%.

#### Recruitment

Subjects will be recruited from the inpatients or outpatients in the Endocrinology Department of Shenzhen TCM Hospital as well as local communities through recruitment advertisements or online promotion. The applicants and main members of the department have been well trained, with rich experience in clinical and experimental research. The Endocrinology Department of Shenzhen TCM Hospital has undertaken multi-stage clinical and basic research projects, and advanced experimental equipment as well as abundant outpatients and inpatients, which jointly ensure the source of patients, technical force and research conditions required by this project. Recruitment was carried out on June 1, 2022, and is supposed to be completed on January 31, 2024.

#### Patient screening

**Fig. 1:**
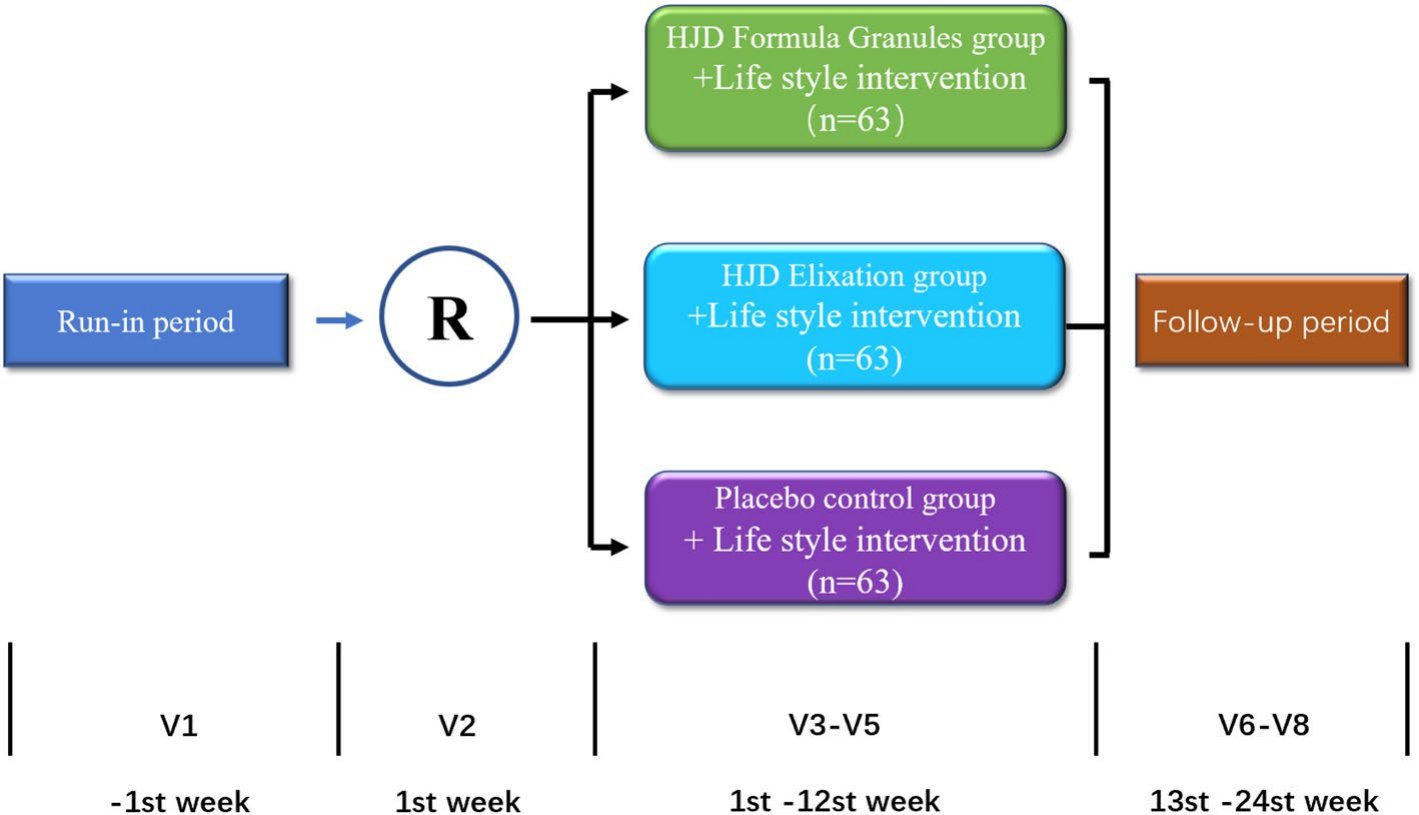
Study flowchart. Members with prediabetes will be enrolled and allocated randomly to one of three groups. All subjects will accept 1 day of screening, and 84 days of intervention and observation. The data is gathered to verify the clinical effectiveness and possible mechanism of Huoxue-Jangtang Decoction (HJD) in the treatment of prediabetes, and to prove the consistency of HJD Formula Granules with HJD Elixation

Prior to the trial, potential subjects will undergo screening at Shenzhen TCM Hospital in accordance with the criteria to determine whether they are eligible for the study. At first, doctors will introduce the study to him/her in as much detail as possible, and carefully answer the doubts of subjects and their families. It is critical that the subjects comprehend the purpose, procedure and duration of the study, as well as the benefits, risks and discomfort that may appear in the course of the research (as shown in Fig. 1).

#### Inclusion criteria

1. Meet the diagnosis of “prediabetes”, namely, the criteria of “impaired fasting glucose” and “impaired glucose tolerance” in the classification of glucose metabolism state proposed by WHO in 1999 [28]. Which means that fasting glucose ≥6.1 mmol/L, < 7.0 mmol/L and/or OGTT 2-hour glucose ≥7.8 mmol/L, < 11.1 mmol/L.
2. ≥18 years old and < 70 years old.
3. HBA1c ≤6.5%.
4. Patients who have not participated in any interventional clinical study or clinical trial in the past 3 months.
5. Patients who are able and willing to sign written informed consent and complete the study.

#### Exclusion criteria

1. Diabetics with clear diagnosis.
2. Women during pregnancy and lactation.
3. Patients with chronic kidney disease (eGFR< 60 mL/min/1.73m^2^ and/or serum creatinine > 133 μmol/L), severe liver dysfunction (any liver enzyme > 3 upper limit of normal, abnormal coagulation function or bilirubin), severe cardiac dysfunction (such as NYHA III or above heart failure, myocardial infarction, and arrhythmia), acute stage of cerebrovascular disease, or malignant diseases that are being treated or have not been fully alleviated or cured.
4. Patients using high-dose probiotics (daily intake >108cFU) in the past 6 months.
5. Patients using glucocorticoids, cytokines that can stimulate the body’s immune system, methotrexate and other immunosuppressants in the past 6 months.
6. Patients systematically using antibiotics in the past 3 months.
7. Patients using local antibiotics in oral cavity within 7 days (such as toothpaste and mouthwash containing antibiotics).
8. Patients with an irregular diet in the past 3 months (the same meal time interval of more than 2 hours).
9. Patients with untreated oral abscess.
10. Patients with oral fungal infection.
11. Patients with oral precancerous lesions or oral cancers.
12. Patients with chronic periodontal diseases requiring long-term treatment.
13. Patients allergic to any of the ingredients in linoleic solution.
14. Persons not suitable for this project according to the researcher because of mental, psychological factors, etc.

### Randomization

Random number tables are provided by statistical professionals and generated by DAS software. The preparation of drug documentation and emergency correspondence is completed by personnel not involved in the clinical trial. Once subjects are recruited in the study, they will be randomly allocated into three groups at a 1:1:1 ratio, namely HJD Formula Granules group (*n* = 63), HJD Elixation group (*n* = 63) and placebo group (*n* = 63). During the trial, drugs will be distributed based on the time sequence of patients’ inclusion for observation and drug number. Drugs shall not be selected, and the drug number will remain unchanged throughout the trial.

### Blinding

This is a double-blind trial in which all the staff, investigators, sponsors and statisticians are blinded. The test drug and placebo are accompanied by an emergency letter with corresponding number, which are kept by the principal investigator. In the serious adverse events, or in need for emergency treatment, the investigator will report to the supervisor and the principal investigator and decide whether to open the emergency letter. The over 20% of opening rate in blind records leakage or emergency letter indicates test failure. After case collection is completed, the database is established and the data is locked, unblinding measures will be performed.

### Withdrawal criteria and management

Medication will be adjusted or terminated if any of the following conditions occur.

1. Severe adverse events related to abnormal blood glucose, such as diabetic ketosis, and hyperosmolar hyperglycemic state.

2. The occurrence of other serious cardiac, cerebral and renal vascular diseases during the test.

3. Severe gastrointestinal reactions.

4. The subjects deviate significantly from test requirements or want to withdraw from the study.

5. Investigators conclude that the subjects are no longer fit for participating in the study.

### Intervention

The main intervention measures include oral HJD Formula Granules, HJD Elixation or placebo in addition to daily lifestyle intervention. The individual personnel will package and label the investigational drug and placebo in accordance with random-number tables and drug blinds. All drugs are concealed in uniform packages with the same label and a 28-day supply. At each visit, the researchers will ask the patient about their medication and count the remaining dose to improve their compliance. Besides, any drug with a clear hypoglycemic effect shall not be used. Previous studies have proved that HJD has the effects of regulating glucose and lipid metabolism, improving inflammation and oxidative stress state, and improving insulin resistance, with good therapeutic effects on type 2 diabetes, metabolic syndrome, and diabetic nephropathy, etc. [25, 29, 30] The dosage of HJD Formula Granules and HJD Elixation is determined by the long-term clinical application experience of our hospital.

The subjects will also be instructed according to the prediabetic lifestyle intervention program contained in *Guideline for the Prevention and Treatment of Type 2 Diabetes Mellitus in China (2020 edition)* [31]. Generally speaking, it mainly includes but is not limited to diet control, exercise and health education, etc. [31]

#### Experimental group

After randomization, 63 subjects will recive HJD Formula Granules and 63 subjects will recive HJD Elixation. The two groups will both take the drug by a single dose of 20 g, 3 times/day, before or after meals, coordinating with lifestyle interventions and continued for 12 weeks.

HJD Formula Granules are provided by Yifang Pharmaceutical Industry. Specifically, the single herb will first be decocted and concentrated to prepare the single herb granules, and then mixed different granules to form the formula granules of HJD. HJD Elixation is prepared by the Pharmacy Department of Shenzhen TCM Hospital. Specifically, the decoction pieces of various herbs are first mixed according to the dosage of HJD, and then form the granules after decocting, concentrating and drying. Whole ingredients of HJD Formula Granules and Elixation are as shown in the Table 2.

**Table 2:** Whole ingredients of HJD Formula Granules and Elixation

Latin binomial nomenclature.	Chinese name	dosage
Rehmannia glutinosa Libosch.	Sheng Di Huang	30 g
*Ophiopogon japonicus* (L. f.) Ker-Gawl.	Mai Dong	20 g
Pseudostellaria heterophylla (Miq.)Pax ex Pax et Hoffm.	Tai Zi Shen	30 g
*Rheum officinale* Bail1.	Da Huang	6 g
Dioscorea opposita Thunb.	Shan Yao	10 g
Astragalus membranaceus (Fisch.) Bge.var.mongholicus (Bge.)Hsiao	Huang Qi	30 g
*Carthamus tinctorius* L.	Hong Hua	15 g
*Prunus persica*(L.)Batsch.	Tao Ren	10 g

#### Control group

The placebo control group (*n* = 63) will take the placebo by a single dose of 20 g, 3 times/day, before or after meals, coordinate with lifestyle interventions and continued for 12 weeks. The Chinese herbal medicine placebo contains pharmaceutical excipients, natural pigments, natural flavoring agents, and granules in the same amount as the test drugs. Its appearance, shape and taste are similar to HJD Formula Granules and HJD decoction Elixation, but it does not have efficacy.

### Outcome measurements

#### Sample collection, storage, analysis and destruction

Researches should communicate with subjects before sample collection, obtain consent of subjects, and sign informed consent. Subjects are forbidden to eat, drink, brush and floss teeth 12 hours before sample collection.

1. Blood collection: 5 ml blood sample should be collected from the median cubital vein at 8:00 a.m. when participants are fasting for more than 8 hours.
2. Saliva collection: Samples will be collected at 8:00 a.m. when participants are fasting for more than 8 hours. Non-irritating methods will be employed to collect saliva samples which should flow naturally into 50 ml EP tube instead of expectorating until 10 ml saliva is collected.
3. Supringival plaque samples collection: Researcher should use sterile curettles to scrape the plaque of subjects’ upper and lower anterior teeth, premolars and buccal side of molars, and place the supringival plaque samples in 1.5 mL EP tubes.
4. Intestinal flora collection: Researcher should collect 2-5 g of fresh and naturally discharged stool samples (the middle part of the stool) with fecal DNA collection tube, avoid mixing with urine or other substances, and replace the clean 5 ml centrifuge tube cover and tighten the collection tube again.

Samples should be placed in − 80 °C refrigerator immediately after collection, and avoid repeated freezing and thawing.

After each sample is analyzed, it will undergo harmless treatment according to the requirements of laboratory waste.

#### Observation index and laboratory examination

During the visit, investigators will arrange the subjects to complete the corresponding examination according to the study progress. Except for physical examination, electrocardiogram and other routine examination, the 3-alternative forced-choice procedure (3-AFC) [32] will be used to detect the threshold of linoleic acid (LA). Subjects in each group will be also guided to complete a modified questionnaire [33] to determine their dietary preferences during the previous 3 months. Saliva and stool samples collected during the study will be analyzed for metabonomics. Specific methods are as follows:

1. Complete physical examination: Specific examination items include general condition (height, weight, BMI, and abdominal circumference), respiratory, cardiovascular, abdominal, skin, head and neck, lymph nodes, thyroid, musculoskeletal (including spine and limbs) and nervous system examination.
2. Routine biochemical tests: Specific tests include blood routine, urine routine, routine stool test, fasting blood glucose (FBG), 2-hour postprandial blood glucose (2hPG), fasting C-peptide, 2-hour postprandial C-peptide, fasting insulin, 2-hour postprandial insulin, hemoglobin A1c (HbA1c), liver function, renal function, and blood lipid. The above tests will be completed by the medical laboratory of Shenzhen TCM Hospital.
3. Oral LA threshold determination: The threshold will be tested by 3-AFC. The solutions used in the test take deionized water as solute, then add 5% edible grade acacia gum and antioxidant 0.01% EDTA, mix thoroughly with blender for 2 minutes, add 5% edible grade liquid paraffin, mix well again, add edible grade LA, and prepare concentration gradients of 0.02, 0.06, 1,1.4, 2, 2.8, 3.8, 5, 6.4, 8,9.8, 12,20 mmol/L LA solution respectively. Blank control solution does not contain LA. All solutions are prepared on the same day and then stored at 4 °C for later use. Subjects should fast for at least 8 hours before testing.

**Fig. 2:**
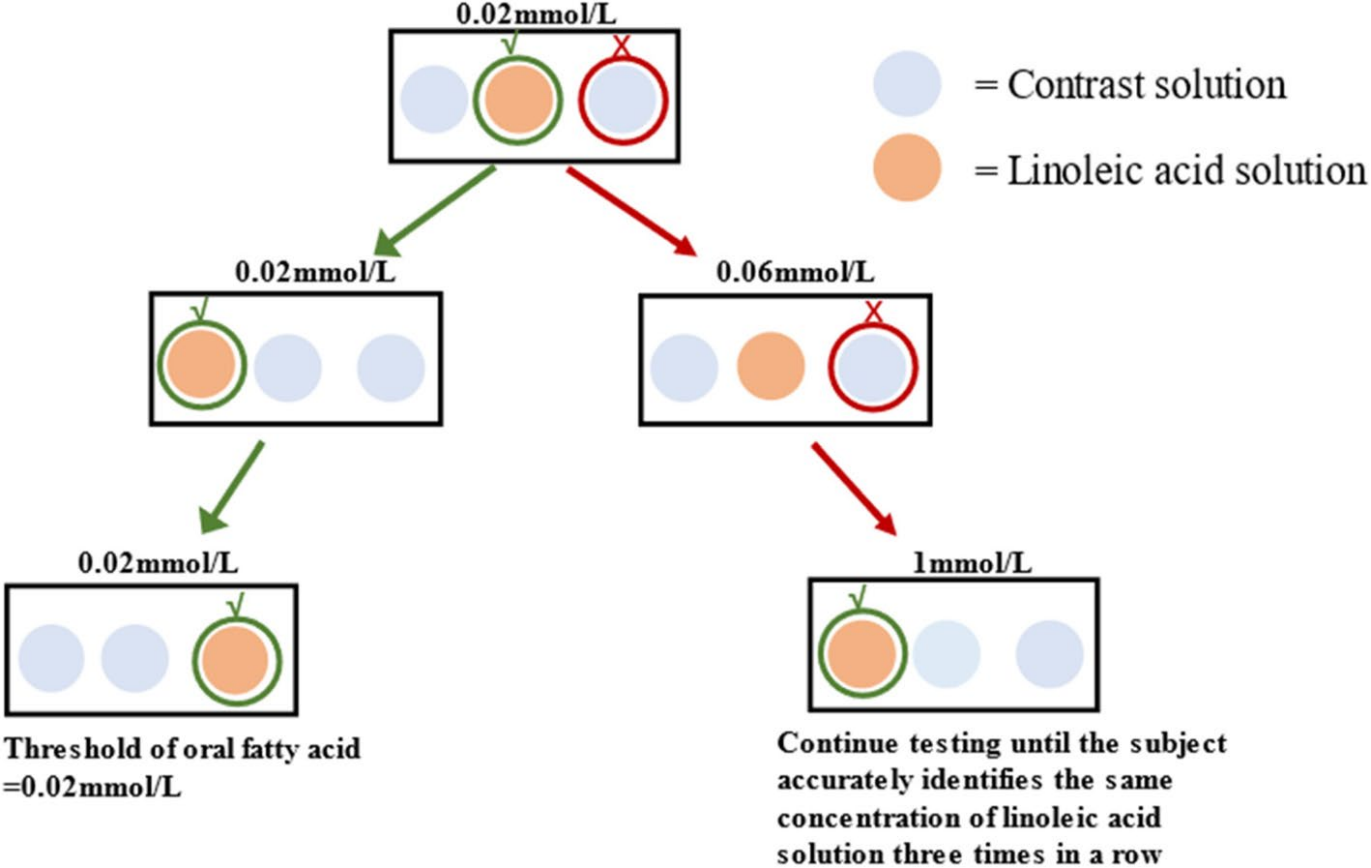
Schematic diagram of 3-alternative forced-choice procedure for detection of oral linoleic acid threshold

During the test, the room should be lit red to avoid visual interference, and the subjects ware nose clips to avoid olfactory interference. At the beginning, each concentration of LA is divided into groups, and each group is lined with 3 small containers, one of which contains different concentrations of LA solution, and the other two are blank control solutions. Starting with the lowest concentration group, subjects are asked to attempt the solutions to identify the LA solution in each of the three containers. At the end of each attempt, they should spit out the solution at the end of each attempt and gargle with deionized water. If the subject is identified incorrectly, he/she will be asked to try the next group with a higher concentration of LA solution. If the subject is identified correctly, the test will be repeated another two times with the same concentration of LA solution. If the subject successfully identifies the same concentration of the solution three times consecutively, this concentration will be the subject’s oral LA-threshold (As shown in Fig. 2).

(4). Dietary preference assessment: Dietary preferences will be evaluated by the questionnaire of dietary status in the last 3 months. (As shown in Table 3).

**Table 3:** Modified dietary preference questionnaires

1. What do you think of your daily food intake? (A) overeating (B) just enough (C) not enough 2. Won’t you feel full until you eat enough? (A) often (B) occasionally (C) feeling full soon before eating enough 3. Do you like rich food? (A) very (B) average (C) not very much (D) very not 4. How often do you eat sweets? (A) often (B) sometimes (C) not often (D) not at all 5. What’s your taste preference? (A) sweet (B) sour (C) bitter (D) spicy (E) salty (F) others 6. What are your diet preference? (A) oily food (B) salty food (C) spicy food (D) light food 7. Which cooking oil do you use most often? (A) vegetable oil (peanut oil, soybean oil, blend oil, or rapeseed oil, etc.) (B) animal oil (lard or butter, etc.) 8. Please recall whether you have eaten any of the following foods in the past 3 months and estimate the average frequency of the intake of these foods. Tick the box that matches your actual intake situation							
Time	≥3 times a day	1–2 times a day	5–6 times a week	3–4 times a week	1–2 times a week	1–3 times a month	≤1 times a month
Type							
Animal skin (fat or offal)							
Cream products (chocolate, cake, bread, or ice cream, etc.)							
Nuts (peanuts, walnuts, or sesame, etc.)							
Fried food (fried dough sticks, French fries, buns, or barbecue, etc.)							
Processed meat (canned meat, ham sausage, or bacon, etc.)

(5). Saliva and intestinal microbiota testing: After thaweding saliva and stool samples, total DNA is extracted using commercial bacterial community DNA extraction kit. After the DNA quality inspection, PCR amplification, product purification and PCR product quantification, 16-second high-throughput sequencing is performed using HiSeq 2500. Bioinformatics analysis is then conducted: After quality assurance of the raw data, the data are de-chimerized and clustered using USearch software. Reads are first sorted according to their abundance from large to small, and obtain OUT (Perational Taxonomic Units) by standard clustering with 97% similarity. Each OUT is thought to represent a species. Following that, Reads from each specimen are extracted at random, and the correlating OUT sequence is extracted. Then, using Qiime software to draw the alpha diversity index dilution curve, and select sensible extraction parameters based on the dilution curve. Use Qiime software to analyze the OUT obtained after extraction. First, a Read is extracted from OUT as a representative sequence. Each OUT will be classified by RDP method to compare the representative sequence with 16S database. After classification, the OUT abundance table is generated based on the number of sequences in each OUT, and the successive analysis is conducted on the basis of the table.
(6). Saliva and intestinal metabolite group detection: After thawing, the saliva and stool samples are mixed, centrifuged, mixed with water of DSS internal standard, centrifuged at low temperature and high speed and detected in a nuclear magnetic tube. 1H-NMR free induction decay (FID) signal will be imported into Chenomx NMR suit (Version 8.0, Chenomx, Edmonton, Canada), and then act Fourier transform, phase adjustment, and baseline correction automatically. Dss-d6 peak (0.0 PPM) is adopted as the chemical shift standard for the whole spectrum, and the spectrum peak shape (CSI) is adjusted by inverse convolution operation. The concentration and peak area of DSS-D6 are used as standards based on signal information in the 1H-NMR spectrum (such as chemical shift, peak shape, half-peak width, and coupling splitting score), and the signal of sample spectrum is compared and analyzed one by one in combination with Chenomx’s own database to obtain the metabolites and the corresponding absolute concentration value. Metabolomics data will be processed using R language, and perform principal component analysis (PCA) and partial least square analysis (PLS-DA) followed by metabolic pathway analysis.
(7). Electrocardiogram examination: Electrocardiogram (ECG) is required during the stage of screening. After the subject has rested for at least 5 minutes, the 12-lead ECG should be performed and recorded on the CRFs as “normal” or “abnormal”.

#### Primary outcomes

The primary outcome is to verify the clinical efficacy and possible mechanism of HJD in the treatment of prediabetes, and to prove the consistency of HJD Formula Granules with HJD Elixation.

It specifically includes the change value of HBA1c, FBG, 2hPG prior to and following treatment.

#### Secondary outcomes

The secondary outcomes are to evaluate the comprehensive effect of HJD Formula Granules and HJD Elixation, specifically including:

1. Oral LA threshold determination.
2. Dietary preference assessment.
3. Changes of body weight and BMI.
4. Time of blood glucose reaching standard.
5. Changes of fasting C-peptide and 2-hour postprandial C-peptide.
6. Changes of cytokines such as CRP, PCT, IL-6 and Adiponectin.

#### Exploratory outcome measure

The exploratory outcomes are to evaluate the effects of HJD Formula Granules and HJD Elixation on oral and intestinal microflora, blood lipid, liver function, and urine function, specifically including:

1. Detection of Salivary and intestinal microbiota and metabolite group.
2. Blood lipid: TG, TC, LDL-c, HDL-c, Apo-A and Apo-B.
3. Liver function: ALT, AST, GGT, albumin and bilirubin.
4. Urine function: BUN, serum creatinine, eGFR, Uric Acid.

#### Safety capability evaluation

Safety capability evaluation refers to evaluating the tolerability and safety of HJD Formula Granules and HJD Elixation, specifically including:

1. AEs/SAEs.
2. Vital signs.
3. Laboratory inspection.
4. ECG.

### Data management

In order to facilitate the subjects to complete the whole study, they will be reminded at each visit point by phone or text message to come to the hospital for relevant examination. All data generated by subjects during the study shall be recorded in the CRFs in detail and truthfully.

With informed consent, uniformly trained investigators will gather data in compliance with the researchers’ manual and Standard Operating Procedure (SOP) formulated in advance. During this period, independent monitors review the data in real time to ensure the accuracy and completeness. In order to facilitate the later data check, all data modification should leave traces. In the case of paper CRFs, the original data should not be changed when the record is corrected or the errors are filled in. Instead, additional statements should be used. A line should be drawn on the original error and the correct data should be written next to it. If inputting the date on the electronic database, the database system should have the function of leaving traces to record the data before modification, modification reason, modification personnel and date as well as other information.

After the completion of data entry, data verification and cleaning are required. For the data with missing information, logical contradiction, error or uncertainty, the supervisor will deliver it to the researcher in the form of question list, and the researcher should answer the question. After verification, the database data will be revised. Any problems found during data verification and cleaning should be notified to the supervisor in time and the researchers are required to answer them. All kinds of questions and answers should use the question sheet, including the request for data supplement and review, etc. All query tables, and error data contents and modification results shall be recorded in detail and properly preserved.

The data of this research may be published in medical publications, but we will keep the subjects’ information secret as needed by law, and personal information about the subjects will not be revealed unless required by relevant laws.

### Statistical analysis

Primary, secondary, exploratory end points and safety assessments will be analyzed when the last patient completes the follow-up or drops out of the study. Analyses of clinical data will be performed by the principal investigator including subjects’ demographic, clinical, and laboratory data.

Continuous variables are used to evaluate the number of cases, mean, standard deviation, median, minimum, and maximum for demographic data and other baseline eigenvalues. Frequency and composition ratio are calculated using count and grade data. Descriptive results invole inferential statistical results (*p*-values).

For the analyses of laboratory data and clinical efficacy of drugs, chi-square test or Fisher’s exact test are used for counting data, while ANOVA and T test are used for measurement data. The grade data is analyzed by Ridit and CMH. The ratio or percentage of counting data is expressed, and the measurement data is expressed as mean ± standard deviation. All statistical tests are conducted by two-sided tests, with *P* < 0.05 indicating statistical significance. Multiple imputation will be used for missing data.

### Adverse events

Although no serious adverse events have been reported with this formulation in previous studies, subjects may have vomiting, diarrhea and other adverse reactions because of individual difference.

Specifically, adverse reactions include clinical symptoms (such as nausea, fatigue, dizziness, abdominal pain, and itching), signs (such as jaundice, rash, and fever), diseases and abnormal laboratory tests, etc. Adverse events are classified as “mild”, “moderate”, or “severe” based on severity. All adverse reactions and their incidence, duration, severity and treatment measures, ending, and the relationship between drug and its dosage will be detailed and fully documented in the CRFs. When a patient experiences an adverse event and does not show significant remission after treatment, the researcher will inform the chief investigator and the ethics committee. Then modification or termination will be conducted to ensure the safety and interests of the subject.

### Trial monitoring

The academic committee of Shenzhen TCM Hospital is responsible for reviewing the scientific nature and compliance of the protocol and making recommendations for the continuation or termination of the study. The ethics committee is responsible for reviewing the rationality of the protocol and safeguarding the rights and interests of the subjects. Both members are independent of the sponsors and researchers, and there is no conflict of interest.

## Discussion

The study’s goal is to investigate the effectiveness and difference of TCM decoction and granule in the management of prediabetes, to clarify whether HJD can adjust fat preferences, and oral and intestinal microbiota of prediabetic patients, and subsequently to slow or prevent the transition from prediabetes to diabetes.

Elixation and granule are the two main dosage forms of TCM treatment, which are widely used in clinic. However, there are few placebo-controlled studies on TCM decoction for prediabetes. After treating prediabetic rats with TCM granules, Wang and colleagues [34] found that TCM granules significantly improved body weight and glucolipid metabolism in rats, and increased insulin sensitivity to an effect similar to that of rosiglitazone. Wei [35] also confirmed in a multi-center trial that TCM granules could restore normal blood glucose in 41.8% of prediabetic patients (27.8% in the control group) after 12 months of intervention. However, the follow-up showed that the granules group had weak lasting effect, and there was no improvement in subjects’ HBA1c levels. Therefore, this study expands the function of TCM elixation in the management of prediabetes, and hopes to significantly improve the symptoms and indicators of prediabetes patients.

Eating habits are closely related to people’s life. People often crave fat because it provides more calories and pleasure [36]. However, high-fat diets also lead to weight gain and decreased sensitivity to fat, which further increases people’s intake of high-fat diets [37, 38]. Poor diet quality has been reported to significantly increase the risk of prediabetes (OR: 1.45, 95%CI: 1.29–1.63) [39], and also affect the changes of oral and intestinal flora [40, 41].

Human saliva contains abundant biological information, and the oral flora can reflect various physiological and pathological changes of the human body. Amr and Kornwipa [42, 43] found that the number of species in oral saliva decreased with the progression of diabetes, and that diabetics exhibited the largest pathogenic microbiome. However, the sample size of their researches was small, the changes of oral microflora in prediabetic patients need to be further explored in a larger sample.

In addition, hyperglycemia will lead to abnormal intestinal flora, further destroy the intestinal barrier, and cause damage to glucose metabolism and immune homeostasis. The study confirmed that after 12 weeks of supervised training, prediabetic patients experienced significant changes in their gut microbiota, as well as remarkable improvements in insulin sensitivity and other metabolic characteristics. After excluding the interference of body weight and visceral fat, correlation analysis confirmed a significant association between the two [44]. What’s more, prediabetes patients had increased multiple butyrate-producing bacteria after inserting the gut microbiota of a healthy person, which could convert fiber into short-chain fatty acids, and then released GlP-1 to suppress appetite and promote weight loss through gut–brain neural circuit [45].

In previous studies, we found that HJD has a good therapeutic effect on metabolic diseases [25–27, 29, 30], and have improved the preparation process of TCM placebo which had been applied for a patent [46], providing a corresponding clinical basis for this study. Therefore, we designed this study to further evaluate the effect of HJD Elixation and Formula Granules in treating prediabetes, the results may also discover the effect of HJD in oral LA threshold, dietary preference, saliva and intestinal microbiota of prediabetes patients, thus to further explain its potential therapy mechanism.

## Data Availability

Following the completion of the present research, the datasets used and/or analyzed will be made accessible to the corresponding author upon reasonable request.

## Abbreviations

TCM: Traditional Chinese medicine
HJD: Huoxue-Jangtang Decoction
ADA: American Diabetes Association
CRF: Case Report Forms
3-AFC: 3-alternative forced-choice procedure
LA: Linoleic acid
FBG: Fasting blood glucose
2hPG: 2-hour postprandial blood glucose
HbA1c: hemoglobin A1c
FID: Free induction decay
PCA: Principal component analysis
ECG: Electrocardiogram
SOP: Standard operating procedure
